# West African Fevers

**Published:** 1898-02-26

**Authors:** 


### West African Fevers.

The great interest which just now attaches to all that pertains to our West African possessions makes it desirable that a word should be said about those diseases which give to that part of the world its bad reputation, which, in fact, render it so unhealthy to Europeans that the negro race will probably always remain the tenants—perhaps the owners of the soil.

Fever and dysentery are the main endemic diseases of the West Coast of Africa. Anæmia should be added as being perhaps the most striking result of residence in those unhealthy regions; but although it may be admitted that prolonged exposure to moist heat is in many cases sufficient to produce this condition, it is quite certain that the anæmia is generally the mark left by dysentery and malaria, the destructive impress on the constitution made by those diseases in such cases as they have failed to kill outright.

Malaria is the great curse of the country, all the conditions for its development existing in a high degree. The moisture, the decaying vegetation, the stagnant, marshy soil, and the continued high temperature, all favour the development of those lowly organisms, which by invading the human body give rise to the multiform manifestations of malaria; organisms which already exist in full activity in virgin primeval swamps where man has never been. Partly by knowledge, and partly by a scientific use of the imagination, a conception of the life history of the malaria organism has been built up which is full of interest. According to this it is a parasite, growing naturally on that swarming animal life which flourishes so abundantly in warm and swampy soils, and especially affecting such blood-sucking creatures as the mosquito, by whom its germs are scattered far and wide in soil and water, and wherever these myriads of insects die. "When, then, man intrudes into a malarial district, he may breathe air charged with germs of the disease arising from the soil, to which it has been carried by the mosquito, or he may drink water in the same way. He may thus become attacked with malaria . . . . . and may in turn infect those mosquitos which may bite him; the man taking the place of some animal as yet unknown" as the host of the parasite. ("Twentieth Century Practice.")

It is on the West Coast of Africa that the fevers caused by the attacks of these parasites take on their most malignant form. Instead of the ordinary agues of more temperate climes, we have the "irregular" malarial fevers, fevers which, instead of being intermittent, hardly even remit; fevers which attack so suddenly and cause such profound coma and collapse as to have often been mistaken for sunstroke; or fevers attended with rapid and extensive destruction of blood-corpuscles, the so-called "blackwater fever," forms which occur with especial virulence on the Gold Coast.

The Bight of Benin, the spot around which interest centres so strongly just now, is said to be the most deadly portion of the West Coast. According to Davidson, no European escapes an attack of fever during some period of his stay on the Gold Coast, while it is said that so common is enlargement of the spleen that no white man resides long in Lagos without being so afflicted. Up in the Yoruba country, however, between 600 and 800 feet above the level of the sea, there are times of the year when the weather is cool and pleasant, while at others, although there are tremendous winds and great thunderstorms, the climate is not essentially unhealthy. Even in these higher regions, however, malaria exercises its sway, dominating all other diseases, for high as is the land, patches of malarial soil exist on every hand.

Europeans in the more civilised parts have, to a certain extent, protected themselves from the other great disease, dysentery, by the use of water stored in protected tanks, and by the use of filters. Directly, however, any movement into the interior is made, the chance of dysentery has to be faced, and this danger is especially aggravated when it is a question of moving troops, by the fact that, from the abundant undergrowth, the tracks along which men move are narrow and quickly become foul.

To move masses of men in certain districts in West Africa near the coast, and at certain seasons of the year, is absolutely impossible, while at the best it is to be feared that men who go to these unhealthy regions run greater risks than those who expose themselves to the chances even of a modern battlefield. The comparative healthiness of the troops in the last Ashanti expedition was the result of the most elaborate precautions and a careful selection of the season, but the deaths of Prince Henry of Battenberg and of Major Fergusson, apparently as the result of a simultaneous exposure, showed how virulent malaria may be even at the healthiest season of the year.

